# Potential Early Effect Biomarkers for Ambient Air Pollution Related Mental Disorders

**DOI:** 10.3390/toxics12070454

**Published:** 2024-06-24

**Authors:** Lijun Bai, Kai Wang, Dandan Liu, Shaowei Wu

**Affiliations:** 1 Department of Occupational and Environmental Health, School of Public Health, Xi’an Jiaotong University Health Science Center, 76 Yanta West Road, Yanta District, Xi’an 710061, China; 2 Key Laboratory of Environment and Genes Related to Diseases (Xi’an Jiaotong University), Ministry of Education, Xi’an 710061, China; 3 Key Laboratory for Disease Prevention and Control and Health Promotion of Shaanxi Province, Xi’an 710061, China; 4 Key Laboratory of Trace Elements and Endemic Diseases in Ministry of Health, Xi’an 710061, China

**Keywords:** air pollution, mental disorders, biomarker

## Abstract

Air pollution is one of the greatest environmental risks to health, with 99% of the world’s population living where the World Health Organization’s air quality guidelines were not met. In addition to the respiratory and cardiovascular systems, the brain is another potential target of air pollution. Population- and experiment-based studies have shown that air pollution may affect mental health through direct or indirect biological pathways. The evidence for mental hazards associated with air pollution has been well documented. However, previous reviews mainly focused on epidemiological associations of air pollution with some specific mental disorders or possible biological mechanisms. A systematic review is absent for early effect biomarkers for characterizing mental health hazards associated with ambient air pollution, which can be used for early warning of related mental disorders and identifying susceptible populations at high risk. This review summarizes possible biomarkers involved in oxidative stress, inflammation, and epigenetic changes linking air pollution and mental disorders, as well as genetic susceptibility biomarkers. These biomarkers may provide a better understanding of air pollution’s adverse effects on mental disorders and provide future research direction in this arena.

## 1. Introduction

A mental disorder is identified by a clinically significant disturbance in an individual’s cognition, emotional regulation, or behavior, often linked to distress or impairment in important areas of functioning [1]. Mental health problems are prevalent all over the world, and approximately one in eight individuals worldwide suffer from a mental illness [2]. Mental disorders are the major causes of years lived with disability (YLDs), occupying one in every six YLDs globally [2]. There is a deficit of affordable essential psychotropic medications worldwide, especially in low-income countries, and a great deal of heterogeneity exists in treatment responsiveness and outcomes [3,4]. Early warning for specific risk factors is probably able to reduce mental health loss by avoiding or lowering related exposures. Mental health varies significantly based on the circumstances in which individuals are born, brought up, and living [5]. Together with lifestyle-related factors, environmental exposures contribute significantly to these modifiable risk factors. Increasing evidence suggests that exposure to air pollution is likely to negatively affect the brain and elevate the risk, severity, and duration of mental health conditions across all life stages [6,7].

From conception to twilight, air pollution affects the human body. Outdoor air pollution is an important environmental health problem affecting individuals in low-, middle-, and high-income countries [8]. In addition to contributing to noncommunicable diseases such as cardiovascular disease, respiratory disease, metabolic disease, and cancer, increasing studies have shown that air pollutants affect the health of brain cells as well [9,10]. The neurotoxic effects of air pollution have become an urgent public health concern [11]. The relationship between air pollution and mental health is complex. Both long-term and short-term adverse effects of air pollution on mental health have been reported currently, in which the long-term exposure is mainly associated with increased incidence of mental disorders and the short-term exposure is generally related to acute exacerbation or episodes of mental disorders. Increasing systematic reviews and meta-analyses have evaluated the association between long- and short-term air pollution exposures and depression, anxiety, schizophrenia, and other mental disorders [12,13,14,15,16,17,18,19,20,21,22,23]. Moreover, environmental policies aimed at reducing emissions of air pollution or greenhouse gases can improve mental health [24], which indirectly verified the mental impairment of ambient air pollution. These population-based studies provide preliminary evidence for air pollution-related risk of mental disorders.

Despite the blood–brain barrier’s natural protection, various pathways have been suggested for the direct intrusion of particulate matter (PM) into the brain [25,26,27]. Existing evidence implies the entry and retention of exogeneous particles into the central nervous system, with the observation of exogeneous fine PM (PM_2.5_) in human cerebrospinal fluids and blood [28,29,30]. The olfactory system is also the potential pathway through which PM_2.5_ enters the body via the nasal cavity and is transmitted to the brain via the olfactory cortex [31,32]. The gut–brain axis is another possible pathway where air pollutants can affect brain function by changing the microbiota and intestinal barrier [33,34]. That is, air pollution may affect mental health through direct or indirect biological pathways. However, a systematic review is absent for early effect biomarkers for manifesting health hazards of ambient air pollution on mental health, which can be used for early warning of potential disease risk. In this review, we discussed the potential hallmarks of ambient air pollution-related mental disorders, along with the possible underlying mechanisms.

## 2. Methods

We indexed relevant reviews and research articles from PubMed database with the following search terms: “air pollution”, “particulate matter”, “sulfur dioxide”, “nitrogen dioxide”, “ozone”, “carbon monoxide”, “mental”, “depress*”, “schizophrenia”, “anxiety”, “bipolar disorder” and “manic depressive psychosis”. A total of 2133 articles on ambient air pollutants and related mental disorders published before 8 April 2024 were selected for screening. The eligible studies were selected by reading the literature abstracts and further detailed analyses of the full literature texts.

### Definition of Early Effect Biomarkers of Ambient Air Pollution-Related Mental Disorders

We propose several early effect biomarkers of air pollution-related mental health loss. Ideally, an early effect biomarker is likely to meet the following criteria: (1) it should be obviously altered in response to exposures to air pollution in human studies, (2) it should be experimentally verified that air pollutants are able to change these biomarkers, and (3) there should be evidence from human-based studies that clearly link the alterations to air pollution-related mental disorders. In spite of the relative inaccessibility of functional human brain, direct leakage of secretions and exosomes from the nervous system into the blood and other fluids, direct connections of nervous system with the rest of the body through vagus nerve, and some developmental commonalities and bi-directional interactions between the immune system and nervous system make surrogate molecular markers to be found in peripheral tissues and fluids for the brain [35]. Considering that humans differ substantially in responses to similar environmental exposure [36], individual differences in susceptibility to ambient air pollution may have a genetic basis. In addition to the potential early effect biomarkers defined above, we also discussed possible susceptibility biomarkers of air pollution-related mental disorders. Susceptibility biomarkers are defined as the factors that can increase, or in some cases, decrease the chance of being impacted by air pollution in terms of developing a mental disorder or aggravating an existing mental disorder.

In this review, we highlight the potential early effect biomarkers in different biological pathways, especially in some emerging research fields, to provide a framework to establish the marker complex indicating air pollution-related mental disorders for environmental monitoring and risk assessment on a cellular and organ level.

## 3. Potential Biomarkers

An increasing number of population-based studies have investigated the association of ambient air pollution with mental disorders [37]. In general, oxidative stress/inflammation and epigenetic changes are the two most widely investigated biological mechanisms for the observed epidemiological associations between ambient air pollution and mental health hazards [38,39]. In this review, we mainly discussed the potential markers associated with these two mechanisms.

### 3.1. Biomarkers of Oxidative Stress and Inflammation

Reactive oxygen species (ROS) play essential roles in both normal brain function and the pathogenesis of neurological diseases [40,41]. An oxidative state occurs when prooxidative processes override cellular antioxidant defenses as a result of the redox signaling disruption [42]. For adenosine triphosphate (ATP) intensive neuronal activity, the brain consumes 20% of its basal oxygen budget. Because of higher oxygen consumption, lipid content, and weaker antioxidative defense, the brain is particularly susceptible to oxidative stress [43]. Preclinical and clinical research have suggested that increased production of ROS and depletion of antioxidative defenses contribute to the changes in brain structure and function [44,45]. Oxidative stress has emerged as a major cause of the pathogenesis and progression of various mental disorders, including depression, schizophrenia, etc. [46,47]. Due to the increased oxidative stress, the subsequently activated proinflammatory signaling pathways also contribute to the pathogenesis of mental disorders [48,49]. Altered peripheral cytokine levels have been linked to brain function, depression [50], anxiety [51], schizophrenia [52], and bipolar disorder [53].

Air pollution exposure can induce oxidative stress and inflammation [54], which may further increase the risk of mental disorders [55]. Nuclear factor erythroid 2-related factor 2 (Nrf2), a pivotal transcription factor responsible for mounting an antioxidant defense, has been found to be involved in air pollution-related health impairments [56]. Nrf2 plays a protective role against immunotoxicity by activating the expression of genes participating in detoxification, antioxidant, and anti-inflammatory processes [56]. Nrf2 is also involved in the regulation of neuroinflammation, mitochondrial dysfunction, autophagical disorder, and ferroptosis [57]. Dysregulation of the Nrf2 pathway may contribute to the development of a series of diseases including mental disorders [57,58]. Chu et al. suggest that sub-chronic PM_2.5_ inhalation in mice could activate the Nrf2 pathway leading to enhanced antioxidative stress and the Nrf2 deficiency could strengthen the oxidative stress and activate nucleotide-binding domain and leucine-rich repeat protein 3 (NLRP3) inflammasome, which may contribute to depression-like behavior induced by PM_2.5_ [59]. Antioxidant defenses facilitated by Nrf2 in the BEAS-2B cells exposed to diesel exhaust PM were found through an increase in the expression of Nrf2 [60]. A previous epidemiological study based on elderly subjects with coronary artery disease reported positive associations of exposure to traffic-related air pollution with gene expression in pathways of the Nrf2-mediated oxidative stress [61]. Therefore, a series of continuous processes concerning oxidative stress, inflammation, and neurotoxicity in response to increased ROS may mirror the mental hazards associated with air pollution.

**Figure 1:**
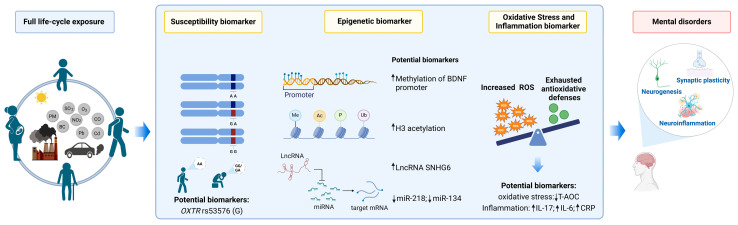
Identified potential early effect biomarkers of mental disorders associated with ambient air pollution exposure.

Several oxidative stress and inflammatory biomarkers have emerged for air pollution–mental disorder associations. For example, a decrease in blood level of total antioxidant capacity (T-AOC) and altered inflammatory cytokines, such as increased interleukin-17 (IL-17), have been found to mediate the potential effects of PM on increased risk of schizophrenia relapse (Table 1) [62,63]. Pottery workers exposed to higher nitrogen dioxide (NO_2_) and sulfur dioxide (SO_2_) showed increased serum levels of 4-hydroxy-2-nonenal and much higher Hamilton Depression Scale scores [64]. Male rats exhibited anxiety- and depression-like behaviors after exposure to simulated vehicle exhaust, which was correlated with lower levels of T-AOC, glutathione, superoxide dismutase activities, and elevated levels of inflammatory markers, such as C-reactive protein (CRP) and tumor necrosis factor-α (TNF-α) in plasma [65]. Through systemic inflammation and particle translocation, real-ambient PM_2.5_ was found to cause microglia activation in mice, featuring excessive release of TNF-α [66]. Among the inflammatory biomarkers in major mental disorders summarized from 43 meta-analyses [67], a few inflammatory factors showed various changes over different mental disorders (e.g., IL-4 increased in bipolar disorder, decreased in suicide, but had no change in depression, autistic spectrum disorder, and schizophrenia). IL-6 and CRP are the two most commonly increased inflammatory factors, and the nerve growth factor (NGF) is the most commonly decreased factor in depression and schizophrenia [67]. On the other hand, exposure to traffic-related air pollution is associated with increased peripheral IL-6, CRP, and TNF-α receptor II (TNFRII) [68]. Inflammatory factors, such as IL-6 and CRP, may also have the potential to serve as early warning indicators for air pollution-related risk of mental disorders (Figure 1). However, the existing evidence for peripheral biomarkers in major mental disorders seems somewhat unconvincing [69,70]. Additionally, peripheral blood data are helpful in the development of clinically informative biomarkers, but the brain is the primary affected target in mental disorders. The establishment of the blood–brain relationship for illustrating the biological mechanisms is still challenging. In a word, identifying oxidative stress and inflammatory markers for air pollution-related risk of mental disorders, especially biomarkers with high specificity, is still a long way to go.

### 3.2. Epigenetic Biomarkers

Despite the genome being relatively stable throughout life, the gene expression is significantly variable [77]. The variability is partially regulated by epigenetic mechanisms, a diverse array of mitotically heritable but reversible molecular changes [78]. Epigenetic changes are increasingly acknowledged as important factors in the etiology and pathophysiology of mental disorders [79,80,81,82,83]. A considerable portion of epigenetic modifications in mental disorders may result from environmental insults and presented as molecular “scars” [80]. Air pollution, one of the major environmental factors, has been linked to epigenetic changes, including DNA methylation, histone modification, and non-coding RNA [84].

#### 3.2.1. DNA Methylations

DNA methylation (DNAm) is an epigenetic process in which methyl groups are added to DNA nucleotides, primarily cytosine and adenine [85]. Methylation is considered to significantly influence the structure and function of DNA through covalent chemical modification. In different genomic regions, distinguishing impacts can be exerted by DNAm levels. DNAm in intergenic regions can inhibit potentially harmful genetic elements that can induce mutation events [86]. Methylation in gene promoter regions generally leads to transcriptional silencing, while gene body methylation can increase gene expression. Abnormalities in global methylation, methylation of specific genes, and associated biological pathways may contribute to the etiology of various mental disorders [85]. Studies across humans and animals have consistently exhibited mental disorder-associated DNAm [87], and the status of DNAm is likely a potential epigenetic biomarker of neuropsychiatric disorders [88]. Air pollution exposure can acutely and chronically induce changes in DNAm across the life course, from an early stage during pregnancy through to old age [89]. On one hand, oxidative species induced by air pollution may downregulate the expression of methionine adenosyltransferase 1A (MAT1A) and lower the efficiency of the one-carbon metabolism pathway, which contributes to a scarcity of the methyl donor S-adenosyl methionine needed to establish and maintain DNAm [90,91]. On the other hand, a lack of maintenance during cell division or by the activity of enzymes (such as ten-eleven translocation methylcytosine dioxygenase) may lead to passive DNA demethylation [89]. Air pollution exposure can enhance DNAm of the ten-eleven translocation methylcytosine dioxygenase, probably generating decreased gene and protein expressions [92]. That is, air pollution is likely to disrupt the balance between effects mediating higher DNAm and removal of methyl groups [89]. Therefore, DNAm can be used to evaluate air pollution-related risk of mental disorders. For instance, previous studies have discovered a correlation between brain-derived neurotrophic factor (*BDNF*) methylation modification and different mental disorders [93,94]. BDNF is a neurotrophic factor crucial for neuronal survival, development, and synaptic plasticity [95].

The human *BDNF* gene includes several untranslated 5′ exons with independent promoters, which can splice with a 3′ coding exon to form a bipartite or tripartite transcript providing various splice variants of *BDNF* mRNA [96]. DNAm in the regulation of *BDNF* gene expression has been identified in cellular and animal models, as well as postmortem brain tissues and peripheral blood tissues of patients with mental disorders [94,97]. A panel study observed that long-term exposure to air pollutants was associated with significantly higher levels of *BDNF* promoter methylation [72] (Table 1). In a cross-over study examining the modification by traffic-related air pollution on the acute effect of exercise on serum BDNF, high concentrations of PM were found to attenuate the increase in serum BDNF brought by cycling [98]. Similarly, in a previous animal study, maternal exposure to PM_2.5_ was found to induce hypermethylation in the *BDNF* promoter and produce detrimental effects on neurobehaviors in multiple generations [99]. However, a panel study based on 34 healthy retirees observed increased concentrations of BDNF after short-term exposure to PM_2.5_ [100]. The discrepancy in existing epidemiologic studies is probably associated with measured and unmeasured factors, such as the study design, population characteristics, sample size, and measurement errors. In clinical studies, antipsychotic treatment has been found to promote many forms of neuroplasticity and increases in *BDNF* expression [101], and increased serum BDNF has been regarded as a candidate biomarker for the successful treatment of depression [102]. The epigenetic changes observed in the *BDNF* gene are considered causal in the pathogenesis rather than merely being an epiphenomenon of mental disorders. Therefore, increased methylation levels in the *BDNF* promoter may serve as a potential indicator for air pollution-related risk of mental disorders (Figure 1). However, there is a discrepancy in reported associations of methylation in the *BDNF* promoter with mental disorders, although most existing studies showed hypermethylation in the *BDNF* promoter in patients with mental disorders [85,94]. Evidence from large population-based and experimental studies is needed to establish the air pollution–methylation relationship in the future.

#### 3.2.2. Histone Modifications

Chromatin is composed of DNA and histone proteins, with the DNA molecule (approximately 150 bp) enveloped by histone octamers. The basic repeating subunit of chromatin, a single nucleosome, is formed by two sets of fundamental histones (H2A, H2B, H3, and H4) [103]. Modifications to the histone landscape can alter the conformation of chromatin within the cell’s nucleus, subsequently further influencing gene expression [104]. Gene expression can be positively or negatively regulated by histones. Chemical modifications that can modify the histone tails include methylation, acetylation, phosphorylation, ubiquitination, small ubiquitin-like modifier mediated modification (SUMOylation), crotonylation, citrullination, and adenosine diphosphate (ADP)-ribosylation, etc. [105]. Histone acetylation at lysine residues is generally associated with the activation of gene transcription. Acetyl groups from lysine residues can be eliminated by histone deacetylases, leading to enhanced ionic interaction of histones with DNA, a tighter DNA pack, and a more condensed chromatin [106]. Furthermore, histone deacetylases seem to be implicated in neuroplasticity, neuronal survival, and cognition, being potential targets of antipsychotic intervention [106], and have been associated with mental disorders [107], such as depression [108], anxiety [109], schizophrenia [110], and bipolar disorder [111]. Significantly, histone acetylation has an adaptive role in stress and depression, for example, acute stressors were reported to induce increased histone acetylation or phosphoacetylation in both mice and rats [112], and continuous social defeat stress was found to be associated with a robust and brief increase, followed by a persistent decrease in the levels of acetylated histone H3 in the hippocampus of mice [113]. The histone deacetylase inhibitor revealed an antidepressant-like effect [113]. The increase in histone H3 acetylation is deemed as a temporary positive adaptation for restoring normal brain function [114].

Histone acetylation plays a pivotal role in mediating the expression and transcriptional activation of many inflammatory genes in response to PM exposure [115,116]. Short-term exposure to traffic-related air pollution has been found to be dose- and time-dependently associated with the changes in *LINE-1 and iNOS* methylation and histone H3K9 acetylation in both the blood and lung tissues of rats [117]. A study on truck drivers and office workers found that short-term exposure to ambient traffic-derived inhalable PM (PM_10_) and black carbon was significantly associated with histone H3 modification in human leukocytes [75] (Table 1). Specific histone H3 modifications have also been identified after exposure to organic chemical compounds and heavy metals in experimental and human studies [76,118,119]. Compared to age-matched controls, histone H3 (such as H3K9K14) in young subjects with schizophrenia is also found to be hypoacetylated, and such hypoacetylation of histone proteins could be reversed by histone deacetylase inhibitors [120]. In the treatment, *BDNF* overexpression was observed to be combined with increased histone H3 acetylation at the *BDNF* promoter [121,122]. Persistent nicotine exposure was found to significantly decrease histone H3 acetylation at the *BDNF* promoter in the ventromedial prefrontal cortex of rats [123]. At present, a few studies have only explored the relationship between short-term exposure to air pollution and histone modification and consistently reported a positive association [75,76] (Figure 1). It is unclear whether histone H3 acetylation may present different changes under short-term and long-term exposures to air pollution, and further investigation is needed to identify the specific histone modifications linking air pollution exposure and mental disorders.

#### 3.2.3. Non-Coding RNAs

Non-coding RNAs (ncRNAs) are another epigenetic modifier susceptible to environmental insults [124]. NcRNAs are RNAs that are transcribed from DNA but not translated into protein, including tRNA, microRNA (miRNA), long non-coding RNA (lncRNA), circular RNA (circRNA), and other types. The roles of ncRNAs, especially miRNAs, are well documented in brain development, stress responses, and neural plasticity [125,126,127]. MicroRNAs (miRNAs) are endogenous ∼23 nt RNAs that play important gene-regulatory roles by pairing to the mRNAs of protein-coding genes to direct their posttranscriptional repression [128,129]. Existing evidence has demonstrated that most miRNAs and lncRNAs are hyperexpressive in the brain [130]. In the brain, miRNAs exhibit specific expression patterns that vary across development stages, cortical layers, and cell types, and can influence a wide array of molecular functions including neurogenesis, neuronal differentiation, circuitry establishment, and synaptic plasticity [131,132,133]. MiRNAs have also been linked to the development of mental disorders including schizophrenia, depression, and bipolar disorder [134]. Despite the discrepancy in specific miRNAs in the blood and brain, significant similarities in gene expression are reported between whole blood and multiple central nervous system tissues, on a transcriptomic level [135]. Whole blood gene expression is fairly tightly linked with several important brain regions in neuropsychiatry, such as amygdala, prefrontal cortex, and whole brain [135]. Circulating miRNAs have been used as effective diagnostic and therapeutic biomarkers for patients [134,136]. Significant changes in cellular murine miRNA expression have been found after exposure to ambient PM near an abandoned uranium mine, and miRNAs from cerebrovascular endothelial cells show similarity to serum-derived miRNAs [137]. Additionally, exosomes are a class of extracellular vesicles derived from endocytosis, abundant in miRNAs, released by cells, and accessible in biofluids, such as saliva, urine, and plasma. Exosomes can serve as mediators of near- and long-distance intercellular communication in health and disease and affect diverse aspects of cell biology [138], participating in processes such as synaptic plasticity, neuronal stress response, cell-to-cell communication, and neurogenesis [139]. Because exosomes can cross the blood–brain barrier, miRNAs in the peripheral exosomes may reflect ongoing neural processes [140]. This cell-to-cell communication facilitated by circulating extracellular miRNAs enables remote neurotoxicity of inhalational exposure to air pollution [141,142]. Circulating exosomes have been found to mediate lung-to-brain crosstalk and lead to brain injury in experiments [143]. Exosome miRNAs have also often been used as an indicator for air pollution-related health effects [142,144,145]. For example, a randomized, crossover study based on 35 healthy college students in Shanghai, China found that 271 exosome miRNAs (212 upregulated and 59 downregulated) significantly changed after exposure to traffic-related air pollution [144]. A cross-sectional study found that short-term exposure to PM_10_ was linked to an increased release of exosomes but downregulated exosome miRNAs, such as miR-218-5p and miR-143, among overweight/obese subjects [74] (Table 1). Meanwhile, low-level expression of miR-218 in the medial prefrontal cortex is a consistent characteristic of depression and changes in miR-218 levels (both upregulation and downregulation) specifically in the medial prefrontal cortex correlate with the expression of miR-218 in blood [146]. MiR-143 is a critical miRNA involved in schizophrenia development by targeting some major genes contributing to the onset of schizophrenia, including *ERK5*, *ERBB3*, *HK2*, and *PKCε* [147]. Thus, several specific miRNAs may be the potential novel early effect biomarkers of air pollution-related mental health impairments (Figure 1). However, animal experimental evidence for the effects of air pollution on dysregulated exosome miRNAs is scarce. A chain of evidence from in vivo and in vitro exposure experiments is urgently needed.

In addition to miRNAs, lncRNAs are also a class of possible biomarkers for air pollution-related mental disorders [148,149,150]. LncRNAs can bind to DNA, RNA, and protein to exert many functions [148]. MiRNAs play a pivotal role in post-transcriptionally regulating protein-coding genes through mRNA cleavage, direct translational repression, and/or mRNA destabilization. LncRNAs can function as miRNA sponges by diminishing the regulatory effects of miRNAs on mRNAs. Large and diverse amounts of lncRNAs have been found in the brain involved in the regulation of important biological processes of the central nervous system [151]. Differential expressions of exosomal lncRNAs following air pollution were observed in human-based studies [71]. Specifically, downregulated lncRNA SNHG6 was found in healthy adults after exposure to high air pollution compared with exposure to low air pollution [71] (Table 1). SNHG6 has also been suggested as an appropriate marker for schizophrenia [152]. Meanwhile, lncRNA SNHG6 is elevated along with the depression-like behaviors in mice, and lncRNA SNHG6 knockdown alleviates the depression-like behavior [153]. However, the existing evidence for the mediating effect of lncRNA SNHG6 in linking air pollution with mental disorders is relatively insufficient, and potential lncRNA biomarkers are worth further discussion.

### 3.3. Susceptibility Biomarkers

Increasing studies suggest that genetic susceptibility can modify the association of air pollution exposure with human health outcomes, including mental disorders [154,155,156,157,158,159]. Genetic susceptibility to the adverse effects of air pollution is generally characterized by a greater response to air pollution due to the presence of certain genetic markers, putting individuals at higher risk. Identifying susceptible genetic markers can provide a basis for further understanding of biological mechanisms and discerning susceptible populations. Rs53576 (G/A), a single nucleotide polymorphism (SNP) located in intron 3 of the oxytocin receptor (*OXTR*) gene, has been associated with individual differences in social behaviors and psychological health, including depressive symptoms [160], suicide attempts [161], affectivity, and emotional loneliness [162]. The human *OXTR* gene is located on chromosome 3p25, spans 17 kb, and contains four exons and three introns [163]. Although the A allele of rs53576 of the *OXTR* gene is likely related to a higher risk of developing mental disorders [160,161,162], the G allele carriers may be more susceptible to air pollution-related mental disorders. A previous study in 86 healthy Chinese preschoolers suggested that the G allele in *OXTR* rs53576 may be a risk factor for social impairment caused by PM_2.5_, with children with GG/GA genotypes being more susceptible than children with the AA genotype [73] (Table 1). Rs53576 polymorphism in the *OXTR* gene is found to be associated with striatal dopamine transporter availability in healthy subjects, and G carriers of this polymorphism could be more susceptible to environmental influences since the negative association of plasma oxytocin level with the striatal dopamine transporter availability was only observed in the G allele carriers of *OXTR* rs53576 [164]. Dysfunction of the dopamine system is a key factor in the pathophysiology of depression and schizophrenia [165,166,167], and environmental factors may play a significant role in dopaminergic dysregulation [167]. Exposure to diesel exhaust has been found to activate dopaminergic neurotoxicity in rats [168]. Dopamine levels in various brain regions of mice, such as the striatum, could also be changed by prenatal exposure to diesel exhaust [169,170]. Decreased dopamine turnover in the striatum and nucleus accumbens, an index of dopamine neuronal activity, has also been observed after exposure to prenatal exposure to diesel exhaust in mice [171]. The above studies suggest that *OXTR* rs53576 may modify the potential effects of ambient air pollution on mental health through dopaminergic dysregulation, and individuals with different genotypes of rss53576 (GG/AG vs. AA) showed differential associations between exposure to air pollution and mental disorders. G allele carriers may present a greater biological sensitivity as well as a greater stress reactivity in response to air pollution exposure (Figure 1). In view of the paucity of existing evidence, more evidence from both population-based and animal-based studies is needed to identify more potential susceptibility biomarkers of air pollution-related mental health loss.

## 4. Conclusions and Prospects

The occurrence and development of mental disorders have been linked to environmental stimuli. Ambient air pollution is increasingly recognized as an emerging issue for brain health [9]. The existing evidence for air pollution-related mental health loss is relatively insufficient compared with the evidence for respiratory and cardiovascular diseases, however, studies focusing on air pollution and mental disorders are growing. These available studies provide preliminary information for early warning biomarkers that can characterize the adverse effects of air pollution on mental health. These biomarkers are related to oxidative stress and inflammation, and epigenetic and genetic mechanisms. Our review suggests that several oxidative stress and inflammatory biomarkers, such as peripheral TAC, IL-17, IL-6, and CRP, may be possible non-specific early warning indicators for air pollution-related development or progressive aggravation in mental disorders. Several epigenetic modifications are potential mental disorder-related markers associated with air pollution, including DNAm levels of certain genes (such as *BDNF*), histone modification, and ncRNAs. Several potential genetic markers of susceptibility, such as G allele carriers of *OXTR* rs53576, are likely to modify air pollution-related mental disorders. However, the existing studies for early warning biomarkers of air pollution-related mental disorders are still insufficient, and heterogeneity in the study population and study design may weaken the reliability and generalizability of the existing study results. Associations reported in current epidemiological studies are probably attributed to direct or indirect causation, and the observed correlation can only suggest possible links, not causation [172]. The convincing causal links between air pollution exposure and effect biomarkers observed in the existing studies need further verification, considering that these available studies are mostly observational based on cross-sectional or panel study design. This review may help explicit the research directions in this field and highlights the need for future well-designed longitudinal research linking air pollution exposure, promising biomarkers, and risk of mental disorders together in the population, as well as in vivo and in vitro experiments for verification and mechanism investigation.

## Figures and Tables

**Table 1:** Summary of major epidemiological studies on the association of ambient air pollution with changes in biomarkers linked to mental health in recent years.

Reference	Air Pollutants	Study Design	Population	Major Exposure Metric	Outcome	Results	Study Area
Lane et al. [68] (2023)	Traffic related air pollution	Cross-sectional	Community-based participants	1-year	Inflammatory biomarkers	Variance (*p*-value) for the association between traffic-related air pollution and inflammatory biomarkers: 0.76 (<0.01) for IL-6; 0.59 (0.02) for CRP; and 0.55 (0.02) for TNFRII.	Boston, Malden and Somerville, MA, USA
Du et al. [71] (2023)	PM_2.5_, UFP, BC, NO_2_, and CO	Cross-over	College students	4 h	Exosomal lncRNAs	Differentially expressed lncRNAs (high air pollution vs. low air pollution): SNHG6 (FC = 6.31, FDR value = 2.42 × 10^−15^).	Shanghai, China
Huang et al. [72] (2022)	PM_1_, PM_2.5_, PM_10_, NO_2_	Panel study	Community-based participants	3-year	Methylation in *BDNF* promoter	Percent changes in methylation level at *BDNF* promoter for the 95th percentile of air pollution concentration against the threshold concentration: NO_2_: 43.25% (95%CI: 13.10%, 73.40%) for average methylation; PM_1_: 128.29% (95%CI: 43.27%, 213.31%) for average methylation; PM_2.5_: 104.22% (95%CI: 34.34%, 174.10%) for average methylation; PM_10_: 108.71% (95%CI: 36.60%, 180.81%) for CpG2 methylation.	Shijiazhuang, China
Sun et al. [73] (2022)	PM_2.5_	Cross-sectional	Preschoolers	30-day	Changes in the urinary acetic acid at different *OXTR* rs53576 genotypes	Absolute changes (μg/mg Cr) in urinary acetic acid per 1 μg/m^3^ increase in PM_2.5_ at different *OXTR* rs53576 genotypes: AA: −11.608 (95% CI: −21.685, −1.530); GG: −16.631 (95% CI: −26.949, −6.314); AG: −28.587 (95% CI: −36.078, −21.096).	Suzhou, China
Wei et al. [62] (2022)	PM_2.5_, PM_10_	Panel study	Stable male schizophrenia patients	8-day	Oxidative stress biomarkers; schizophrenia relapse risk: evaluated by early signs scale (ESS), ESS-anxiety/agitation (ESS-A), ESS- excitability/disinhibition (ESS-D)	Absolute changes in cytokines per 10 μg/m^3^ increase in PM_2.5_: CAT: −0.039 (95% CI: −0.060, −0.017) U/mL; SOD: −1.258 (95% CI: −1.975, −0.541) U/mL; T-AOC: −0.076 (95% CI: −0.126, −0.026) mmol/l. Regression coefficient for mediation of cytokines on ESS score changes associated with per 10 μg/m^3^ increase in PM_2.5_: T-AOC: 0.019 (95% CI: 0.002, 0.040) for ESS-A; T-AOC: 0.013 (95% CI: 0.002, 0.030) for ESS-D.	Hefei, China
Gao et al. [63] (2021)	PM_2.5_, PM_10_	Repeated measure	Stable schizophrenia patients	0–5-day	Immune cytokines; schizophrenia relapse risk: evaluated by ESS and ESS-A	Absolute changes (pg/mL) in cytokines per 10 μg/m^3^ increase in PM_2.5_ at lag0: IL-6: 0.335 (95% CI: 0.045, 0.625); IL-17: 0.191 (95% CI: 0.144, 0.238); IL-2: 0.071 (95% CI: 0.030, 0.112); IL-12: 3.158 (95% CI: 1.719, 4.597); IFN-γ: 1.168 (95% CI: 0.645, 1.691). Regression coefficient for mediation of cytokines on ESS-A score changes associated with per 10 μg/m^3^ increase in PM_2.5_ at lag0: IL-17: 0.202 (95% CI: 0.012, 0.392).	Hefei, China
Pergoli et al. [74] (2017)	PM_10_	Cross-sectional	Overweight or obese subjects	0–7-day	EV count and miRNA content of EVs	Percent changes in EV count per 10 μg/m^3^ increase in PM_10_ at lag 1: EV count: 3.5%, *p* = 0.0001; miR-218-5p: −4.20% (95% CI: −1.87%, −6.47%); miR-143-3p: −2.75% (95% CI: −0.04%, −5.39%).	Lombardy, Italy
Zheng et al. [75] (2017)	PM_10_, PM_2.5_, BC, elemental components	Repeated measure	Truck drivers and office workers	14-day	Histone H3 modification level	Percent changes in histone modifications per 1 μg/m^3^ increase in 14-day average PM_10_ exposure: H3K27me3: −1.1% (95% CI: −1.6%, −0.6%); H3K36me3 levels: −0.8% (95% CI: −1.4%, −0.1%) in all participants Percent changes in histone modifications per 1 μg/m^3^ increase in 14-day average BC exposure: H3K9ac: 4.6% (95% CI: 0.9%, 8.4%) in office workers; H3K36me3: 4.1% (95% CI: 1.3%, 7.0%) in truck drivers.	Beijing, China
Cantone et al. [76] (2011)	Metal-rich PM	Cross-sectional	Steel workers	3-day	Histones H3K4me2 and H3K9ac	Standardized regression coefficient for changes in histone modifications associated with an increase equal to the difference between the 90th and 10th percentiles of nickel and arsenic exposures: H3K4me2 Nickel: 0.16 (95% CI: 0.01, 0.3); Arsenic: 0.16 (95% CI: 0.03, 0.29); H3K9ac Nickel: 0.24 (95% CI: −0.02, 0.51); Arsenic: 0.21 (95% CI: −0.06, 0.48).	Brescia, Italy

Abbreviations: BC: black carbon; *BDNF*: brain-derived neurotrophic factor; CAT: catalase; CO: carbon monoxide; CRP: C-reactive protein; EV: extracellular vesicles; FC: fold change; FDR: false discovery rate; H3K9ac: histone 3 lysine 9 acetylation; H3K4me2: histone 3 lysine 4 demethylation; IL: interleukin; IFN-γ: interferon-γ; NO_2_: nitrogen dioxide; OXTR: oxytocin receptor; PM_1_: particulate matter ≤ 1 µm in diameter; PM_10_: particulate matter ≤ 10 µm in diameter; PM_2.5_: particulate matter ≤ 2.5 µm in diameter; SOD: superoxide dismutase; TNFRII: tumor necrosis factor-α receptor II; UFP: ultrafine particles.

## Data Availability

No new data were created or analyzed in this study. Data sharing is not applicable to this article.
